# Misdiagnosis of an Odontogenic Infection as a Skin Lesion: The Diagnostic Dilemma

**DOI:** 10.7759/cureus.66024

**Published:** 2024-08-02

**Authors:** Anushka Jain, Amit Reche, Soumya Singhai, Priyanka Paul, Khushee Raisoni, Komal Agrawal

**Affiliations:** 1 Dentistry, Sharad Pawar Dental College and Hospital, Datta Meghe Institute of Higher Education and Research, Wardha, IND

**Keywords:** extraoral draining sinus, periapical healing, misdiagnosis, root canal therapy, chronic periapical abscess

## Abstract

Extraoral sinus tracts of endodontic origin might be confused for a variety of dermatological conditions. Differential diagnosis of this clinical condition plays an essential role in providing appropriate clinical care because misdiagnosis is the most prevalent cause of prolonged therapy and healing failure. As a result, every cutaneous sinus structure affecting the face or neck should be investigated for dental issues. Its diagnosis can sometimes be difficult until the treating clinician examines the potential of a dental cause. Once an appropriate diagnosis has been established, definitive treatment, consisting of root canal therapy or tooth extraction, to remove the primary source of infection is a straightforward and successful operation.

## Introduction

The chronic apical abscess is an inflammation in the periapical area that eventually progresses and forms pus at the apices of the root. The sinus tract is a common finding since this lesion is symptomless. It is an indication of persistent infections of the dental tissue that allow infections and pus to drain [1,2]. The sinus tract follows the path of least resistance, which can appear either intraoral or extraoral. Factors that can determine the position of the sinus tract are the thickness of the alveolar bone and overlying soft tissue, distance from the root apex, outer cortical bone, and shape of the bone [3-6]. The vestibule of the mouth along the buccal alveolar plate is the most common site for the emergence of the oral sinus [7]. Commonly, extraoral sinuses appear anywhere on the face, neck, cheek, chin, mandibular angle, and sometimes the floor of the nose [8]. Because they resemble cutaneous lesions, we commonly encounter misdiagnosis and mismanagement. As this lesion appears on the skin's surface, it can be misdiagnosed due to the absence of any dental symptoms for a variety of disorders, including topical cutaneous infection, growth hair, osteomyelitis, tuberculosis, actinomycosis, and congenital midline sinus of the upper lip [9,10]. In this case report of persistence, extraoral cutaneous sinus is described, which was previously misdiagnosed and was treated by antibiotics, which led to delayed healing later as the dental origin was diagnosed and the treatment of endodontic therapy was planned. The healing of the periapical lesion was evident both clinically and radiographically at the 12-month follow-up.

## Case presentation

**Figure 1:**
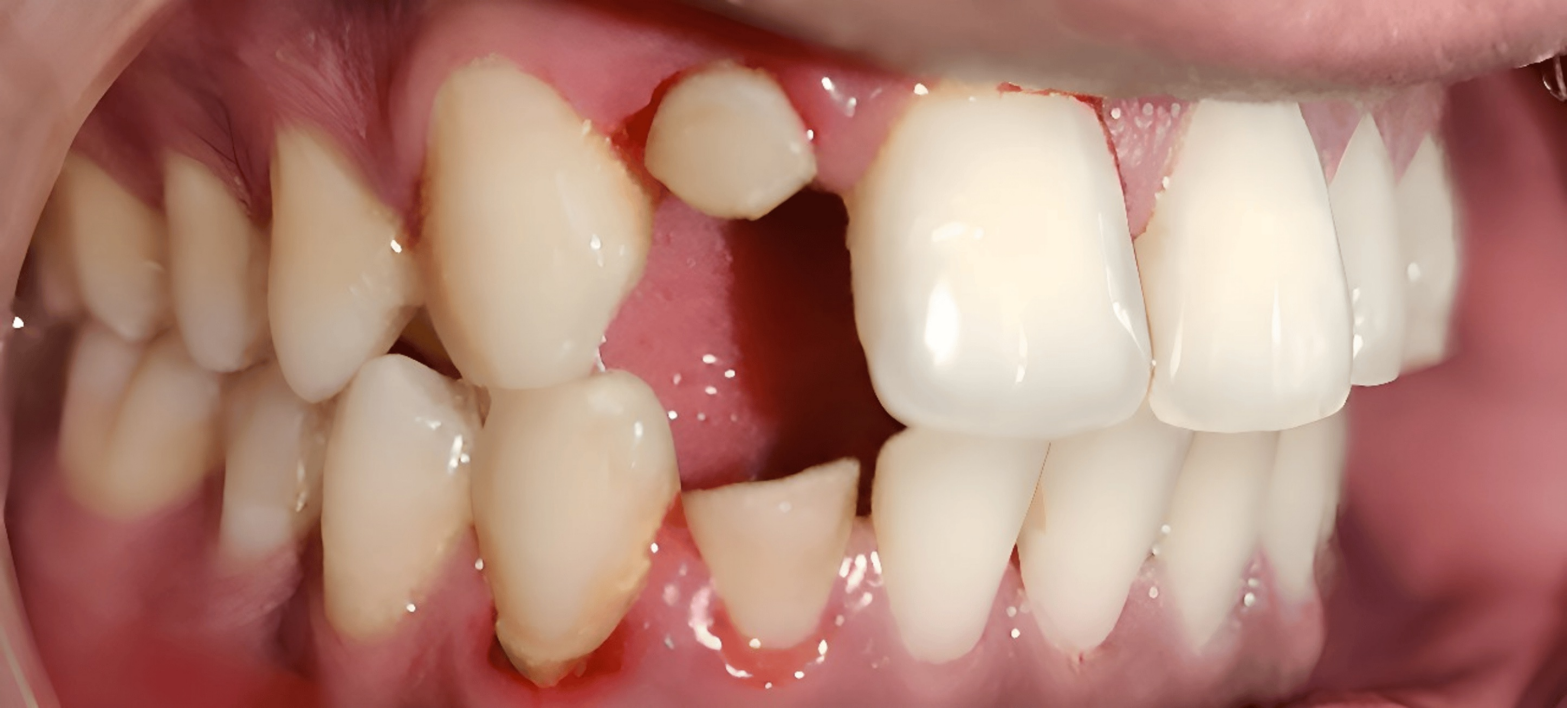
Preoperative clinical image of a fractured lateral incisor

A healthy 19-year-old male patient visited the Conservative and Endodontics Department complaining about a pimple on the face. He gave a history of trauma one year from a road traffic accident, his lower and upper lateral incisors on the right side, and the fractured lateral teeth (Figure 1).

**Figure 2:**
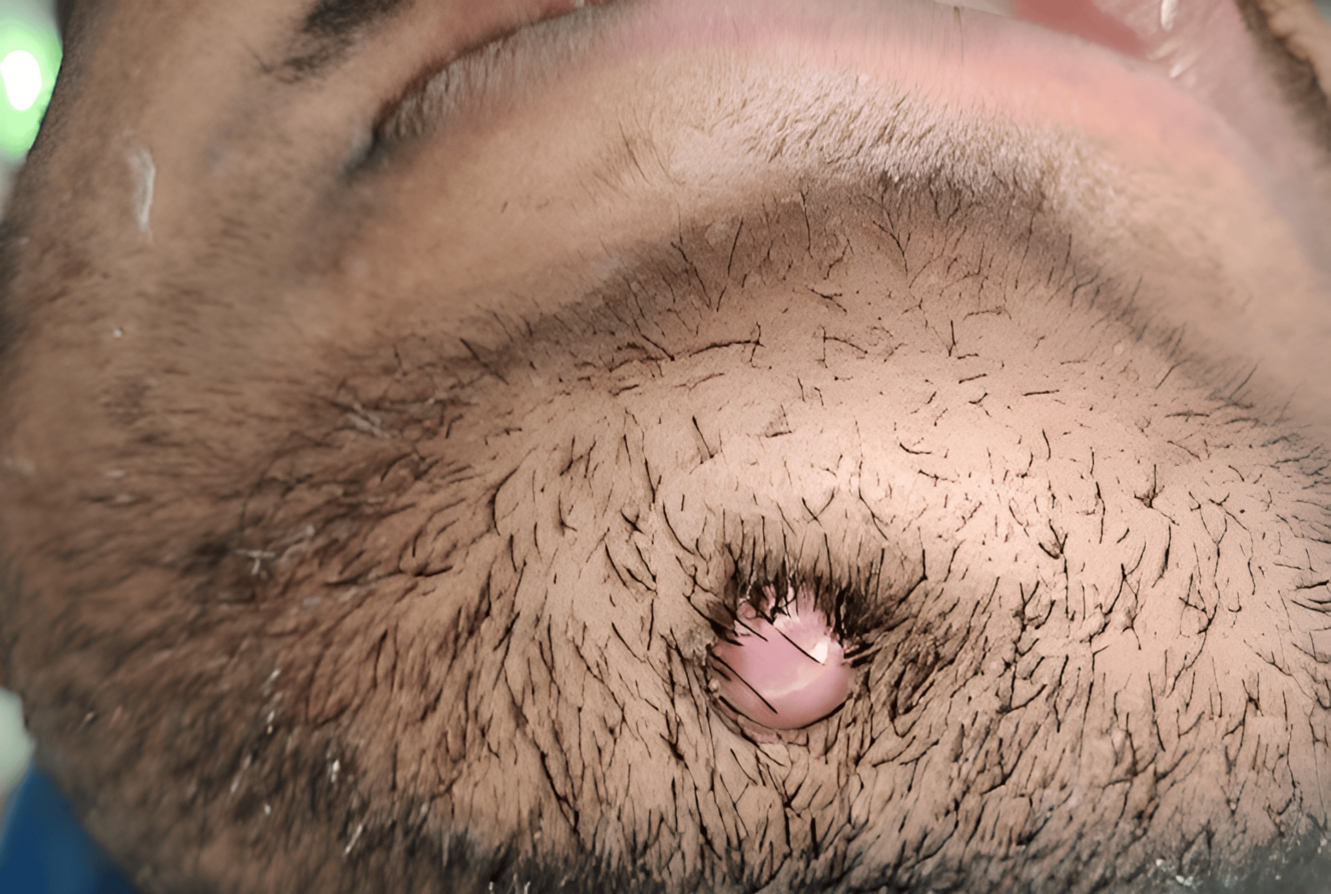
Extra-oral draining sinus in the chin region

He was very anxious and wanted to know the cause of the skin lesion present on his right cheek. During history taking, the patient revealed that the lesion had been present for two months (Figure 2).

**Figure 3:**
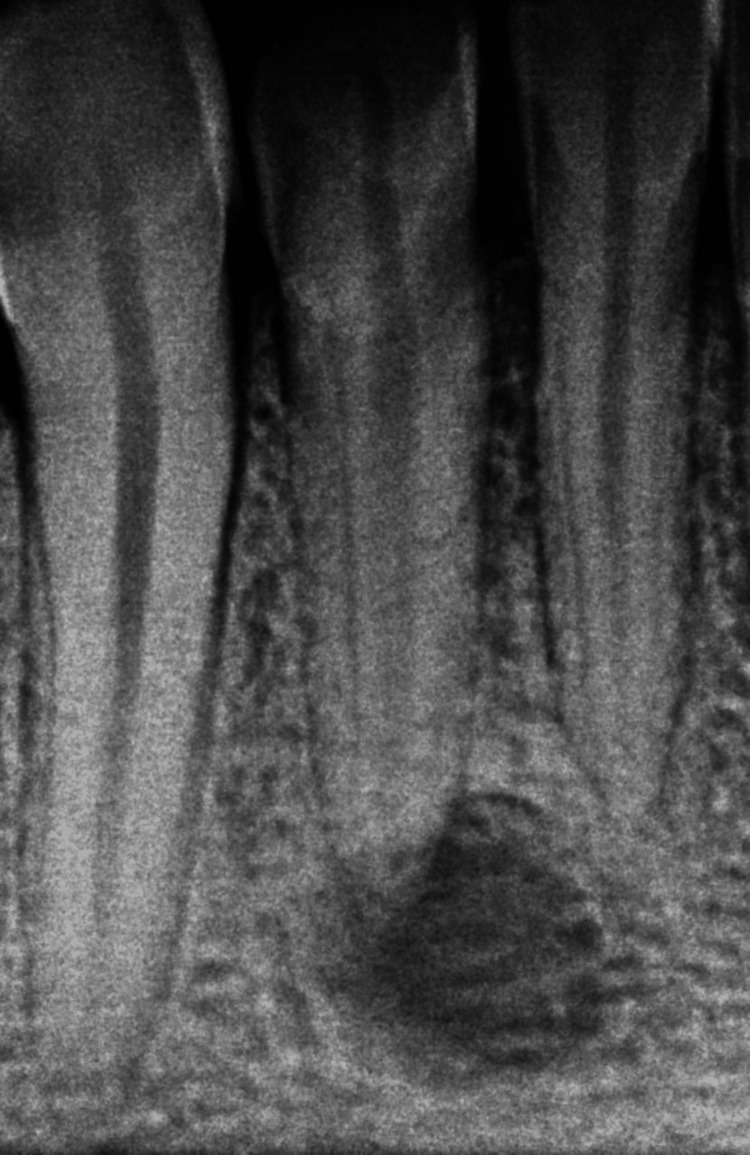
Preoperative intraoral periapical radiograph

Like any other skin disease that appeared on the skin, this patient was also treated by a general physician for several weeks with antibiotics. The patient explained the type of pain was intermittent; there was no history of night pain, and continuously, there was draining pus through the lesion. During the examination, a gutta-percha point of 25.4% was used to confirm the path of the sinus tract, which revealed that the culprit's tooth was the lower-right lateral incisor. During the preoperative examination, an intraoral periapical radiograph was taken, which revealed a diffuse area of bone rarefaction (Figure 3).

**Figure 4:**
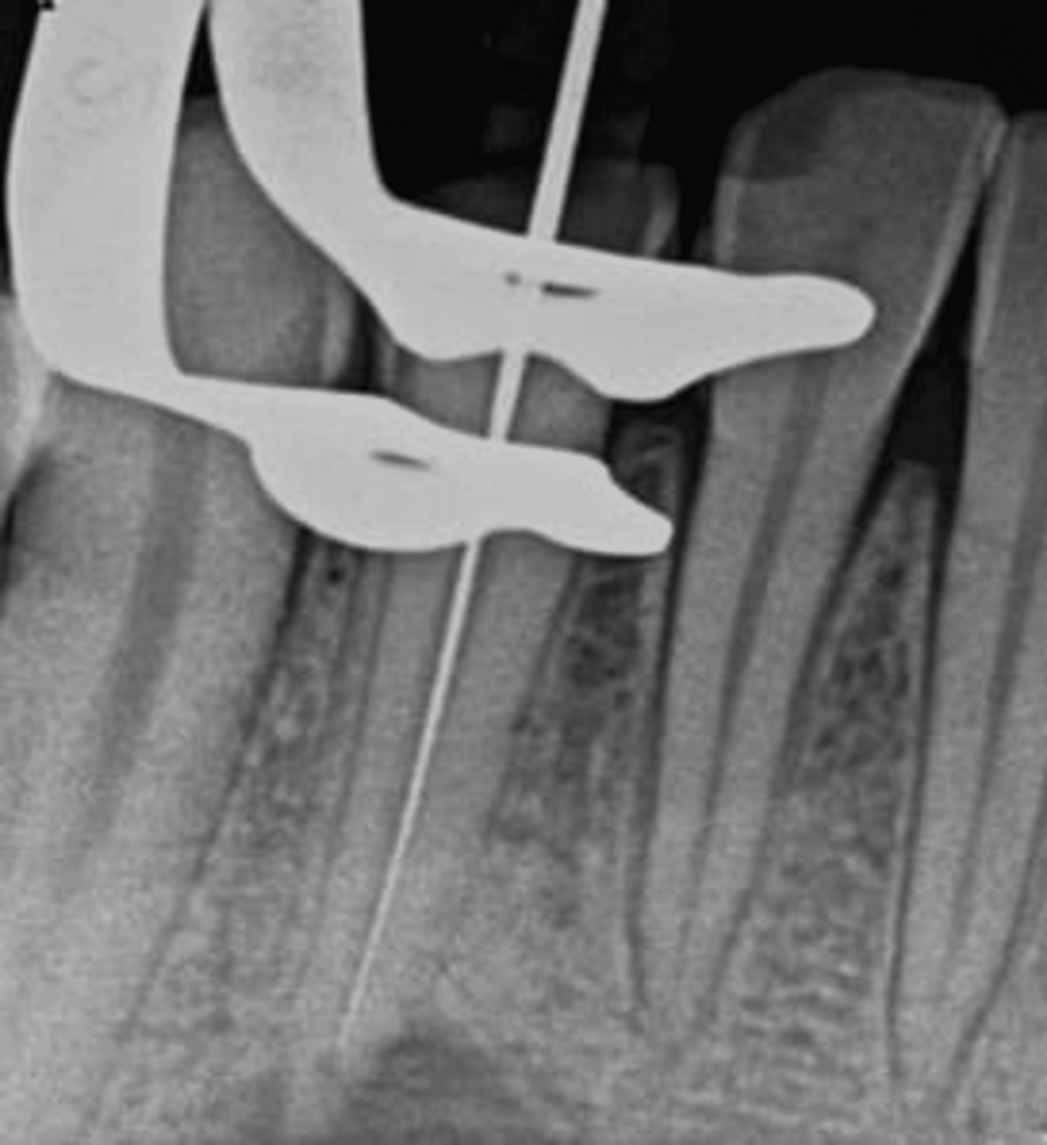
Working length determination

While performing percussion tests, the tooth was asymptomatic and unresponsive to thermal and electric pulp vitality tests. After clinical and radiographic examination, a chronic periradicular abscess with a cutaneous sinus tract as a diagnosis was concluded to be associated with the lower-right lateral incisor tooth. Informed consent was obtained from the patient, and after that, the root was treated with root canal treatment. Access opening was done, and a No. 15 (Mani) K file was used, for negotiating the canals. The working length was determined using an apex locator and a radiograph (Figure 4).

Irrigation was done with saline coming out of the extra-oral sinus, confirming that the lesion was in the sinus tract with an odontogenic origin. As saline was coming out, extra-oral chlorhexidine was preferred as an irrigant during the cleaning and shaping of the canal due to the risk of apical extrusion of sodium hypochlorite solution. After the enlargement, up to 20.4% of the rotary file (NeoEndo Flex) was used. A paper point of 20.4% was used to dry the canal, and intracanal calcium hydroxide was given for one week.

**Figure 5:**
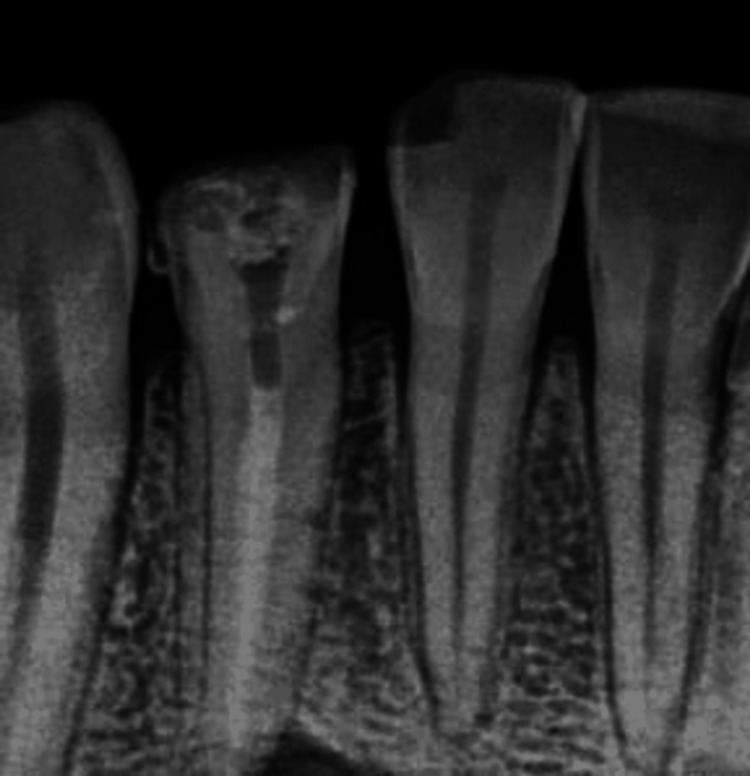
Calcium hydroxide placement for one week

**Figure 6:**
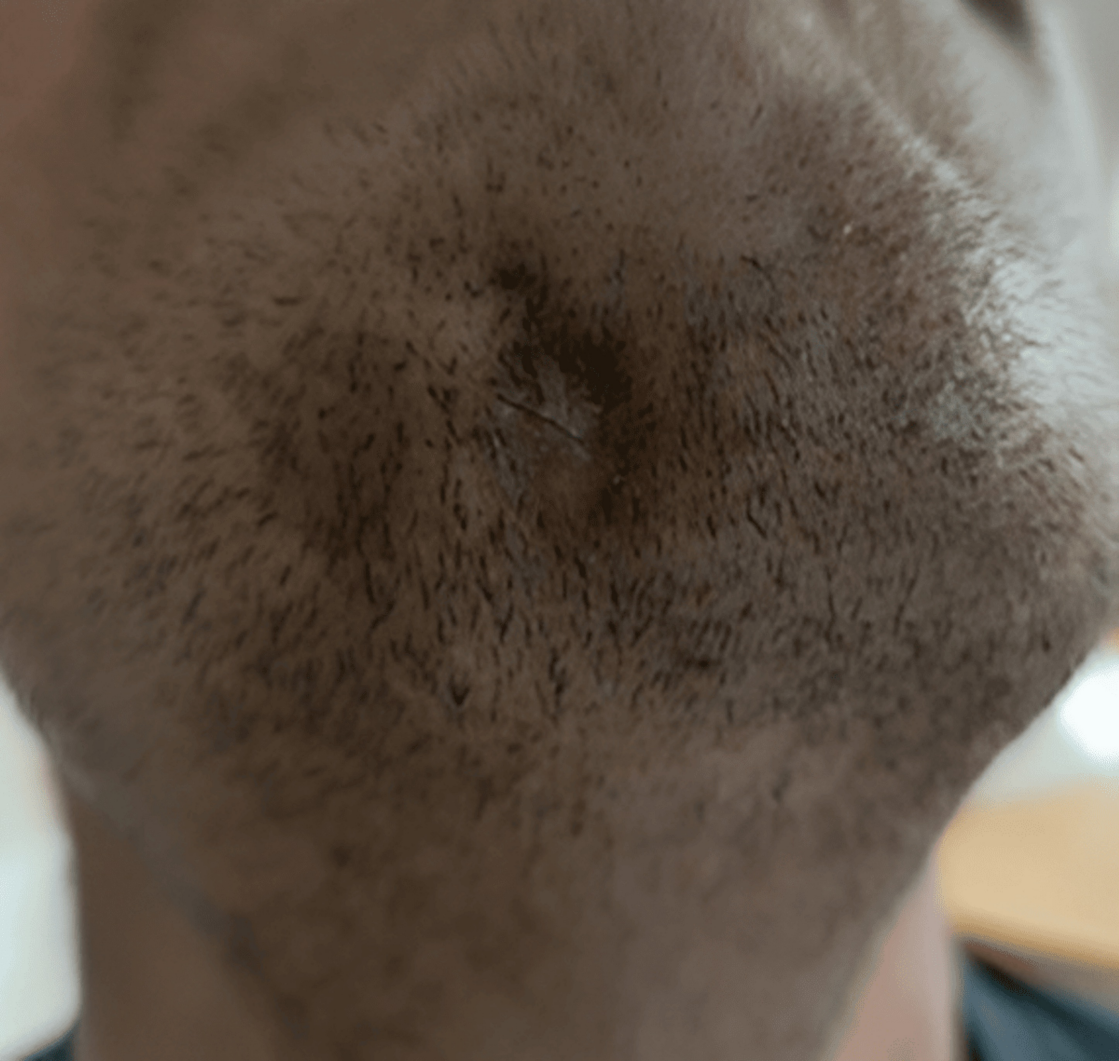
Clinical image of the healing of the extra-oral sinus

After one week, calcium hydroxide was removed, and irrigation was done with sodium hypochlorite, which was left in the canal for 15 minutes to avoid extrusion that could occur due to irrigation. The canal was irrigated with saline, and the final rinse was carried out with chlorhexidine. To ensure complete disinfection, chlorhexidine was left in the canal for 10 minutes. The canal was dried and obturated with a single-cone obturation technique using a bioceramic sealer; a temporary dressing was given. In the third visit after removing the temporary dressing, resin-modified glass ionomer followed by a composite was done (RMGIC). Root canal therapy of the upper-right lateral incisor was also planned at the subsequent visit. At the 12-month follow-up, clinically on the face area, healing of the extra-oral sinus was evident with nominal scarring (Figure 5). During the radiographic examination, after one year, healing of the periapical lesion was evident (Figure 6).

## Discussion

Extraoral sinus tracts are usually misdiagnosed because they mimic cutaneous skin diseases. When diagnosing a discharging lesion in the face or neck region, a dental etiology must be considered the most important factor [11]. The etiology of a lesion can be dental trauma, deep dentinal caries, periodontal problems, inadequate restorations, infection of the periapical area, or surrounding the apices of the root as the consequence of pulpal disease [12]. A differential diagnosis could be a foreign body reaction, a fungal infection of the skin, squamous cell carcinoma, osteomyelitis, or pyogenic granuloma [13]. The recurring condition of slowing advances via the alveolar bone, along the course of least resistance, until it passes through the cortical plate of the jaws and produces a subperiosteal abscess [14]. When the teeth's apices in the maxillary region have a superior muscle attachment, those in the mandibular region have inferior muscle attachments. Far more probable chances of cutaneous sinus tract occur [15]. There is an 80% chance of draining the mandibular abscess onto the submandibular or chin region.

Odontogenic cutaneous sinus tracts appear clinically as the infection spreads in the surrounding soft tissues. In this case report, calcium hydroxide is used as an intracanal medicament. Ricucci et al., in their study, found that periapical lesion sizes of 1-5 mm in diameter have a success rate of 86.6%, and if the size of the lesion is larger, the success rate is 78.2% [16]. Non-surgical endodontic therapy is the choice for most cases of periapical lesions. The follow-up period to access the healing of the periapical lesion must be at least six to 12 months after the endodontic therapy. It is observed that 88% of the time, healing is evident in most cases after 12 months, whereas in only half of the cases, healing is evident after six months. Specific infections that occur on the skin may present clinical signs that are very similar to those of odontogenic infections, and this a cause for concern. Skin lesions such as pustules, furuncles, foreign-body lesions, squamous cell carcinoma, basal cell face carcinomas, and granulomatous disorders may resemble draining sinus tracts of dental origin, but they are not the same. In this case report, nonsurgical root canal therapy resulted in the closure of the extraoral sinus tract, confirming that the skin lesion was dental in origin [17].

## Conclusions

The extraoral sinus has healed significantly, indicating that the root canal therapy with antibacterial irrigants and, after drying of the root canal, the placement of intracanal medicaments is the preferred therapy in these cases. Initially, surgical intervention is not indicated for the involved teeth. Clinical observations indicate a reduction in inflammation and discharge, with the sinus gradually closing. The patient has reported a decrease in pain and discomfort. Continued adherence to the prescribed treatment regimen and regular follow-up visits have been instrumental in achieving these positive outcomes. Overall, the prognosis for complete healing is favorable.
